# A Novel Hepacivirus in Wild Rodents from South America

**DOI:** 10.3390/v11030297

**Published:** 2019-03-24

**Authors:** William Marciel de Souza, Marcílio Jorge Fumagalli, Gilberto Sabino-Santos, Felipe Gonçalves Motta Maia, Sejal Modha, Márcio Roberto Teixeira Nunes, Pablo Ramiro Murcia, Luiz Tadeu Moraes Figueiredo

**Affiliations:** 1 Virology Research Center, Ribeirão Preto Medical School, University of São Paulo,14049-900 Ribeirão Preto, São Paulo, Brazil; marcilio_jorge@hotmail.com (M.J.F.); sabinogsj@usp.br (G.S.-S.J.); felipegmaia@gmail.com (F.G.M.M.); ltmfigue@fmrp.usp.br (L.T.M.F.); 2 MRC-University of Glasgow Centre for Virus Research, Glasgow G61 1QH, UK; s.modha.1@research.gla.ac.uk (S.M.); pablo.murcia@glasgow.ac.uk (P.R.M.); 3 Department of Genetics, Evolution and Bioagents, Institute of Biology, University of Campinas, 13083-862 Campinas, São Paulo, Brazil; 4 Institute of Biomedical Sciences, University of São Paulo, 05508-900 São Paulo, São Paulo, Brazil; 5 Center for Technological Innovations, Evandro Chagas Institute, Ministry of Health, Ananindeua, 67030-000 Pará, Pará, Brazil; marcionunesbrasil@yahoo.com.br

**Keywords:** Viral hepatitis, *Hepacivirus*, *Flaviviridae*

## Abstract

The *Hepacivirus* genus comprises single-stranded positive-sense RNA viruses within the family *Flaviviridae*. Several hepaciviruses have been identified in different mammals, including multiple rodent species in Africa, Asia, Europe, and North America. To date, no rodent hepacivirus has been identified in the South American continent. Here, we describe an unknown hepacivirus discovered during a metagenomic screen in *Akodon montensis*, *Calomys tener*, *Oligoryzomys nigripes*, *Necromys lasiurus,* and *Mus musculus* from São Paulo State, Brazil. Molecular detection of this novel hepacivirus by RT-PCR showed a frequency of 11.11% (2/18) in *Oligoryzomys nigripes*. This is the first identification of hepavivirus in sigmondonine rodents and in rodents from South America. In sum, our results expand the host range, viral diversity, and geographical distribution of the *Hepacivirus* genus.

## 1. Introduction

The *Hepacivirus* genus (*Flaviviridae* family) comprises single-stranded, positive-sense RNA viruses with a genome of ~10 Kb in length that encodes for a polyprotein. This polyprotein is processed co- and post-translationally by the host and viral proteases giving rise to ten proteins [1]. Within the *Hepacivirus* genus there are 14 viral species, named *Hepacivirus A* to *N* [1]. The viruses have a broad host range as they have been described not only in humans but also in horses (*Hepacivirus A*), cows (*Hepacivirus N*), primates (*Hepacivirus B*), and bats (*Hepacivirus K* to *M*). In addition, hepaciviruses have been described in several rodent species (*Hepacivirus E* to *J*) [2]. Among the latter group, hepaciviruses have been identified in rodents from Asia, Africa, Europe, and North and Central America [3,4,5]. The rodent-associated hepaciviruses have been described in *Neotominae* subfamily, represented by Hepacivirus E in deer mouse (*Peromyscus maniculatus*), *Arvicolinae* subfamily, including Hepacivirus F and J found in bank voles (*Myodes glareolus*), and *Muridae* family with Hepacivirus G and H described in brown rat (*Rattus norvegicus*), and Hepacivirus I reported in four-striped grass mouse (*Rhabdomys pumilio*) [3,5,6]. Also, it was recently described new putative hepaciviruses species in midday jird (*Meriones meridianus*), Clarke’s vole (*Neodon clarkei*), northern three-toed jerboa (*Dipus sagitta*), and Mongolian five-toed jerboa (*Allactaga sibirica*), both last species are from *Dipodidae* family [7]. However, hepaciviruses have not been described in sigmodontine rodents or in South America. Here, we report the discovery and characterization of the first hepacivirus detected in South America. This virus was identified in black-footed pygmy rice rats (*Oligoryzomys nigripes*) captured in the northeastern region of São Paulo State, Brazil.

## 2. Materials and Methods

### 2.1. Rodent Samples and Ethical Statements

Serum samples were collected from 647 wild rodents between 2008 and 2013, in a rural area in the northeastern region of São Paulo State, Brazil. Based on morphological features and cytochrome-b gene these rodents were classified in five different species, *Akodon montensis* (199 samples), *Calomys tener* (109 samples), *Oligoryzomys nigripes* (63 samples), *Necromys lasiurus* (252 samples) and *Mus musculus* (24 samples), as previously described [8]. Blood samples were collected by retro-orbital plexus puncture with non-heparinized capillary tubes and then transferred to microtubes. All animal procedures were approved by the Brazilian Ministry of Environment (No. 19838-5), the Ethics Committee for Animal Research of the University of São Paulo (No. 020/2011) and approved by state law (licenses IF-SP/COTEC No. 260108-007.043).

### 2.2. Viral RNA Extraction, Sequencing, and Assembly 

Samples were divided into pools based on species and collection date and prepared as previously described [8,9]. Each pool contains between 18 to 59 serum samples (Table 1). Then, viral RNA of pooled samples were extracted using the QIAamp viral RNA mini kit (Qiagen, Hilden, Germany) as recommended by the manufacturer. The pool contain 20 µL of each individual samples described in Table 1. cDNA synthesis was performed using SuperScript II and random hexamers (Invitrogen, Carlsbad, CA, USA). Subsequently, sequencing was performed using the TruSeq RNA sample preparation kit in an Illumina HiSeq 2500 instrument (Illumina, San Diego, CA, USA) with a paired-end and 150-base-read protocol in RAPID module. The sequencing reads were assembled *de novo* using the metaViC pipeline (https://github.com/sejmodha/MetaViC) as previously described applied to identify a range of other novel viruses [10]. The coverage of viral genome was determined by alignment using Bowtie2 of reads against assembled genome [11]. In addition, it was performed a screening of all reads data of pools described in Table 1 against novel hepacivirus genome recovered in pool 14 using Bowtie2. 

### 2.3. Genome Characterization 

Putative ORFs, molecular weight, and the size of viral genomes were predicted using Geneious 9.1.2 (Biomatters, Auckland, New Zealand). Protein domains were screened using InterProScan [12] and manually compared with those of related hepaciviruses. The viral genome determined in this study was deposited in GenBank (accession number MH370348). 

### 2.4. Phylogenetic Analysis 

Maximum likelihood (ML) phylogenetic trees were inferred using protein alignments of two regions. The first selected region was from amino acid position 1123 to 1156 (NS3 protease), and the second from amino acid position 2536 to 2959 (NS5-RdRp), both numbered relative to Hepatitis C virus subtype 1a (GenBank accession M62321) as previously described [13]. Sequence alignments contained sequences of the virus identified in this study together with 25 sequences obtained from representative members of the *Hepacivirus* genus [1]. The multiple sequence alignment (MSA) was generated using PROMALS3D [14] with manual adjustments. ML trees were inferred with IQ-TREE (version 1.6.0) using the substitution models LG + F + I + G4 (for NS5) and LG + I + G4 (for NS3) with 1000 ultrafast bootstraps [15]. The best-fit models were determined based on Bayesian Information Criterion in ModelFinder [16]. Statistical support for individual nodes of the phylogenetic trees were estimated using the bootstrap value. The phylogenetic trees were visualized using FigTree (version 1.4.2). 

### 2.5. p-Distance Analysis

The evolutionary distances among clades were estimated for the NS3 and NS5 alignments using p-distance values. All ambiguous positions were removed for each sequence pair (NS3 = 3.95% and NS5 = 2.09% of ambiguous positions). Standard error estimations were calculated with the bootstrapping method (1000 replicates) using the MEGA (version 7) [17].

### 2.6. Recombination Analysis

We screened for potential recombination events using an MSA of 25 coding nucleotide sequences. To this end, we used the RDP, GENECONV, Bootscan, MaxChi, Chimaera, SiScan and 3Seq methods implemented in RDP4 [18]. Default settings for all methods were used to identify breakpoints and putative recombination regions. The highest acceptable p-value was set to 0.05 after Bonferroni correction for multiple comparisons. All method-specific program settings remained at their default values.

### 2.7. RT-PCR for Novel Hepacivirus

Viral RNA of individual rodents (*n* = 18) samples were extracted using the QIAamp viral RNA extraction kit (Qiagen, Hilden, Germany) and converted to cDNA using Moloney Murine Leukemia Virus Reverse Transcriptase with random hexamers (Invitrogen, Carlsbad, CA, USA), following the manufacturer’s instructions. Then, we designed primer sets to specifically amplify a 1260 nt-long segment of the NS5 gene of the viruses identified in this study (forward primer: 5′-GGCCTACATGACCCGGCCTG-3′-position 7430 to 7449 nt; reverse primer: 5′-GACCAGTCCTTGCCCCACCAATC-3′-position 8,689 to 8,667 nt) (Figure S1). Subsequently, PCR was performed using Platinum Taq DNA Polymerase high fidelity (Thermo Fisher Scientific, Waltham, MA, USA), following the manufacturer’s instructions. The cycling conditions were: 94 °C for 30 s followed by 35 cycles at 94 °C for 15 s, 60 °C for 30 s and 68 °C for 90 s. Amplicons were visualized by gel electrophoresis in 2% agarose gels. Also, all PCR products were verified by dideoxy sequencing using an ABI 3730 genetic analyzer (Applied Biosystems, Foster City, CA, USA).

## 3. Results and Discussion 

**Figure 1:**
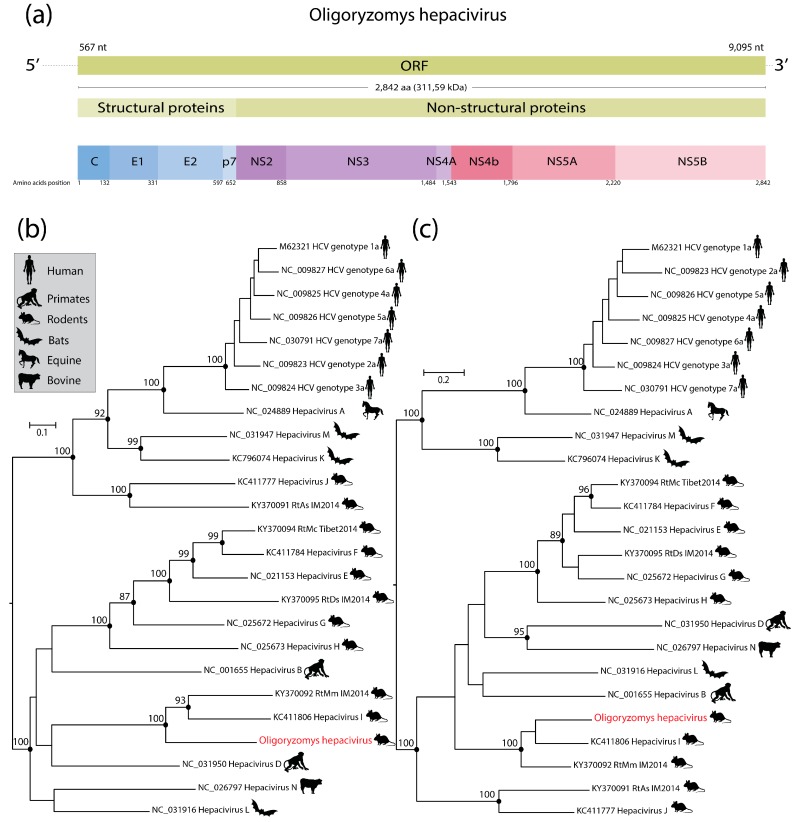
Putative genome organization of the nearly complete genome of *Oligoryzomys hepacivirus* (OHV) (**a**). Maximum likelihood phylogenetic trees showing the evolutionary relationships of OHV with representative members of the *Hepacivirus* genus in NS3 protease (**b**) and NS5 RdRp (**c**). Phylogenies were midpoint rooted for clarity. The scale bar indicates evolutionary distance in numbers of substitutions per amino acid site. Bootstrap values (≥85) of 1000 replicates are shown in main nodes. The OHV sequence generated in this study is highlighted in red color. HCV: Hepatitis C virus; RtAs: Rodent hepacvirus Allactaga sibirica; RtMC: Rodent hepacvirus Neodon clarkei; RtDs: Rodent hepacvirus Dipus sagittal; RtMm: Rodent hepacvirus Meriones meridianus.

A nearly complete hepacivirus genome was identified in a pool of blood samples derived from black-footed pygmy rice rats (*Oligoryzomys nigripes*) that were captured between 2012 and 2013 in the northeastern region of São Paulo State, Brazil. This virus was provisionally designated as *Oligoryzomys hepacivirus* (OHV) to indicate the origin of the host species. The nearly complete OHV genome was 9124 nucleotides in length and presented a typical genome organization similar to members of the *Hepacivirus* genus [1]. The OHV genome was obtained by 2637 reads with a median coverage of 110 (Figure S2). The viral genome contains a unique ORF encoding a single polyprotein with 2842 aa, which is putatively cleaved in ten proteins (Figure 1a) as previously described for rodent hepaciviruses [2]. Also, we obtained the partial 5’ (556 nt) and 3’ (29 nt) untranslated regions (UTRs). BLASTX analysis showed that the OHV polyprotein shared between 48% and 49% amino acid identity with hepaciviruses described in rodents from China (GenBank accession KY370092) and South Africa (GenBank accession KC411806). In addition, after the analysis of fifteen metagenomic datasets described in Table 1, we found 2%–78% of bacteria, 14%–93% of eukaryotic and 3%–59% of virus genomes within contigs assemblies (Table S1). Also, we found reads related to OHV exclusively into the dataset from pool 14.

**Figure 2:**
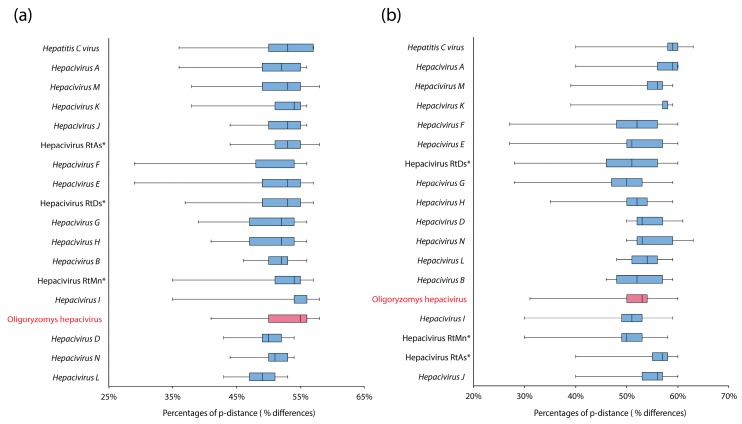
Amino acid p-distances of the OHV and representative members of the *Hepacivirus* genus in NS3 protease (**a**) and NS5 RdRp (**b**). The ends of the box represent the upper and lower quartiles, and the box spans the interquartile range. The median is showed by a black vertical line inside the box and the whiskers are the two lines outside the box that extend to the highest and lowest p-distance. The percentages of p-distance are shown in the *X*-axis and the representative members of *Hepacivirus* genus are shown on the *Y*-axis. The asterisks indicate the unrecognized species by ICTV. The novel hepacivirus is highlighted in red.

Phylogenetic trees inferred from amino acids alignments of two conserved genome regions (NS3 protease and NS5—RNA dependent RNA-polymerase (RdRp) exhibited similar topology, but presented minor and non-bootstrap supported rearrangements of deep branches (Figure 1b,c), as previously reported [13]. However, in both trees OHV formed a monophyletic clade with Hepacivirus I identified in a four-striped grass mouse (*Rhabdomys pumilio*) captured in South Africa [5], and the unclassified hepacivirus from midday jird (*Meriones meridianus*) captured in China (Figure 1b,c). We did not find any evidence of recombination in the OHV genome. OHV exhibited amino acid differences greater than 0.3 to other species of the *Hepacivirus* genus in both regions (Figure 2). In addition, the OHV shared only 45% amino acid identity with Hepacivirus RtMn and *Hepacivirus I*. Based on the species demarcation criteria for this genus by the International Committee on Taxonomy of Viruses (i.e., a novel hepaciviruses must exhibit a p-distance greater than 0.25 between amino acids 1123 to 1566 of the NS3 protein, and greater than 0.3 between amino acids 2536 and 2959 of NS5), we propose that OHV should constitute a new hepacivirus species. 

Almost 30 years after the discovery of HCV, and despite extensive research efforts, the origins of this virus remain unclear [2]. Rodents are hosts of the majority of hepaciviruses, and considering that rodents account for more than 40% of mammalian diversity, it seems very likely that they would be responsible for a significant proportion of cross-species transmission events [2]. Also, the observed phylogenetic trees did not suggest co-speciation events [3,19] thus suggesting that the diversity of rodent hepaciviruses might be a consequence of multiple cross-species transmissions [2]. Therefore, the identification and characterization of novel hepaciviruses might point towards the identification of lurking reservoirs of human disease, and can also aid to elucidate the evolutionary origins of HCV.

OHV nucleic acids were detected by RT-PCR in 11.11% (2/18) of blood samples from *Oligoryzomys nigripes*. The positive samples shared 98% of identity in partial genome of NS5 gene, suggesting two strains of same hepacivirus species. Both positive rodents were sampled on 11 June 2012 in a farm in Batatais town, and none of the animals have presented any signs of diseases. In addition, dilution factors and low viremia may eventually result in undetected hepaciviruses. Our results showed that rodents presented viremia, suggesting that viral replication is likely taking place in these hosts, and further indicates that rodents might act as natural reservoirs for this virus. A previous study showed evidence of hepatotropism in bank voles (*Myodes glareolus*) and suggested that these rodents may clear hepacivirus infections [5]. On the other hand, a novel hepacivirus identified in Norway rats from New York City can establish a hepatotropic persistence infection in rats, which is similar to the hepaciviruses described in humans and horses [20,21]. As our sample collection strategy was based on a capture-release approach, we were unable to obtain liver samples for viral detection in tissues. For the same reason and given the small volume of blood collected, as well as OHV can be as difficult to cultivate as HCV, no isolation attempts or virus rescue trials in animals were made. Therefore, further studies are needed to investigate the pathogenesis of OHV in rodents. Also, some negative samples in our study could be a result of viral clearance as previously reported in bank voles [5]. Thus further studies applying the detection of antibodies against hepaciviruses can be useful to understand this phenomenon.

Most rodent hepaciviruses have been described in members of the subfamilies *Arvicolinae*, *Neotominae* (*Cricetidae* family), and *Muridae* family from Asia, Africa, Europe, and North America [3,4,5]. The fact that we sampled five different species of rodents but detected OHV only in *Oligoryzomys nigripes* suggests a low detection rate or putative restricted host range, which needs to be investigated in larger populations of rodents, and in different geographic regions. On the other hand, the different depth coverage obtained by sequencing in all pools in our study could affect adversely the identification of OHV genome in other pools this study. However, the discovery of OHV expands the geographical distribution of hepaciviruses to South America and its host diversity to the *Sigmodontinae* subfamily, which is one of the most diverse groups of mammals with ~377 species [22]. Our findings provide new information on the host range, diversity, and evolution of this important group of viruses.

## Figures and Tables

**Table 1:** Information of sample pools used in this study.

Pool	Species	N^1^	Collection Date	Number of Reads
1	*Akodon montensis*	55	2008	27,569,342
2	*Akodon montensis*	55	2008	23,439,326
3	*Akodon montensis*	41	2009	16,698,848
4	*Akodon montensis*	48	2012–2013	18,783,944
5	*Calomys tener*	38	2008	27,017,352
6	*Calomys tener*	37	2008	23,972,304
7	*Calomys tener*	34	2009, 2012–2013	15,679,756
8	*Necromys lasiurus*	59	2008	22,989,548
9	*Necromys lasiurus*	59	2008	9,252,300
10	*Necromys lasiurus*	58	2008	18,213,066
11	*Necromys lasiurus*	52	2009	22,210,888
12	*Necromys lasiurus*	24	2012–2013	25,122,228
13	*Oligoryzomys nigripes*	43	2008–2009	15,813,430
14	*Oligoryzomys nigripes*	18	2012–2013	24,796,054
15	*Mus musculus*	24	2008–2009	16,661,928

^1^N: number of individual samples per pool.

## Supplementary Materials

The following are available online at https://www.mdpi.com/1999-4915/11/3/297/s1, Table S1: Percentages of contig assemblies of each pool. Figure S1: Alignment of primer sets region with representative hepacivirus sequences. Figure S2: Mapping of reads against genomes of *Oligoryzomys hepacivirus*.
