# Supplementary material for: A Novel Hepacivirus in Wild Rodents from South America
Source: Viruses. 2019 Mar 24;11(3):297. doi: 10.3390/v11030297 (PMC6466192; doi:10.3390/v11030297)
Supplement: Supplementary file 1 [file viruses-11-00297-s001.zip › Supplementary/Table S1.docx]

**Table S1.** Percentages of contig assemblies of each pool.

| **Pool** | **Eukaryota** | **Bacteria** | **Viruses** |
| --- | --- | --- | --- |
| 1 | 48 % | 40 % | 9 % |
| 2 | 27 % | 50 % | 20 % |
| 3 | 17 % | 78 % | 4 % |
| 4 | 36 % | 42 % | 21 % |
| 5 | 38 % | 28 % | 31 % |
| 6 | 36 % | 46 % | 16 % |
| 7 | 14 % | 26 % | 59 % |
| 8 | 64 % | 21 % | 14 % |
| 9 | 87 % | 5 % | 5 % |
| 10 | 20 % | 76 % | 4 % |
| 11 | 93 % | 2 % | 4 % |
| 12 | 55 % | 23 % | 21 % |
| 13 | 30 % | 67 % | 3 % |
| 14 | 68 % | 27 % | 4 % |
| 15 | 54 % | 30 % | 15 % |
