# Supplementary figures and images for: A Novel Hepacivirus in Wild Rodents from South America
Source: Viruses. 2019 Mar 24;11(3):297. doi: 10.3390/v11030297 (PMC6466192; doi:10.3390/v11030297)

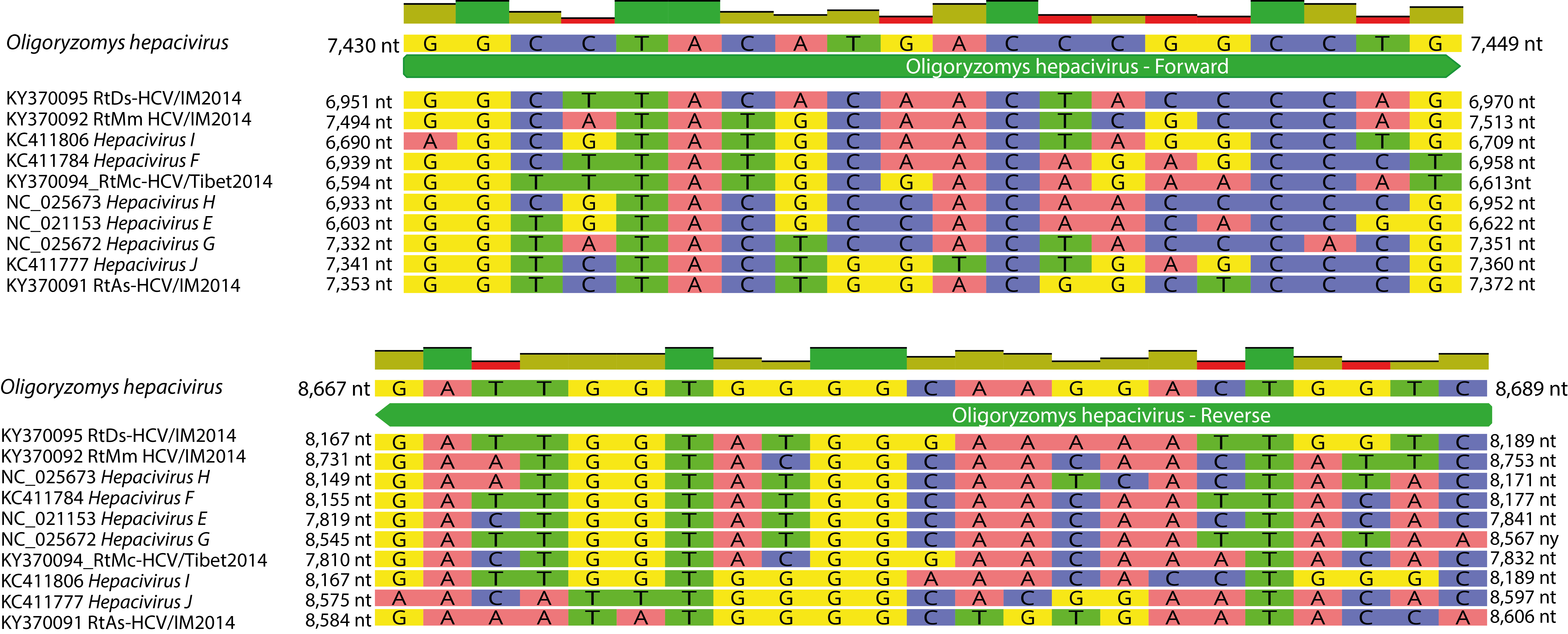

Supplement: Supplementary file 1 [file viruses-11-00297-s001.zip › Supplementary/Figure S1.png]

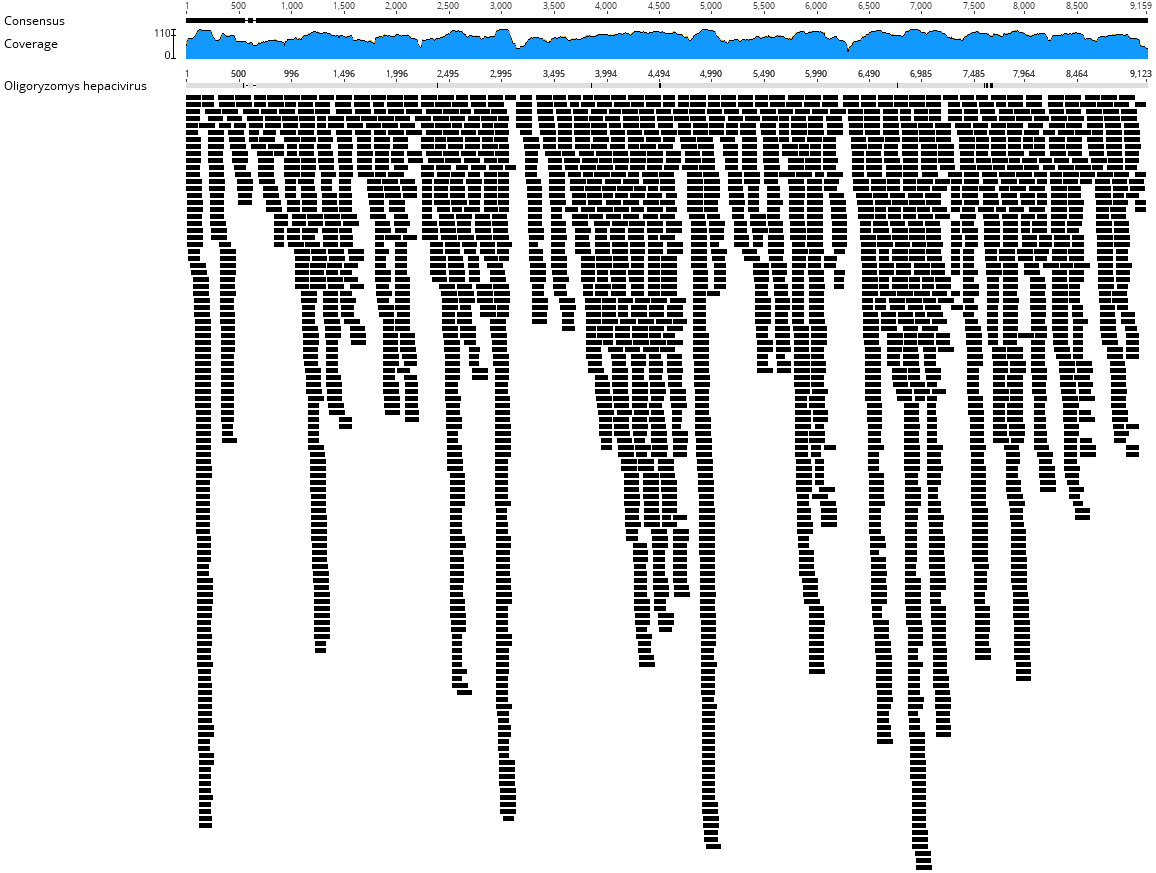

Supplement: Supplementary file 1 [file viruses-11-00297-s001.zip › Supplementary/Figure S2.png]
